# *Streptococcus agalactiae* from Ethiopian pregnant women; prevalence, associated factors and antimicrobial resistance: alarming for prophylaxis

**DOI:** 10.1186/s12941-019-0303-3

**Published:** 2019-01-19

**Authors:** Mucheye Gizachew, Moges Tiruneh, Feleke Moges, Mulat Adefris, Zemene Tigabu, Belay Tessema

**Affiliations:** 1 Department of Medical Microbiology, School of Biomedical and Laboratory Sciences, College of Medicine and Health Sciences, University of Gondar, P. O. Box 196, Gondar, Ethiopia; 2 Department of Gynecology and Obstetrics, School of Medicine, College of Medicine and Health Sciences, University of Gondar, P. O. Box 196, Gondar, Ethiopia; 3 Department of Pediatrics, School of Medicine, College of Medicine and Health Sciences, University of Gondar, P. O. Box 196, Gondar, Ethiopia

**Keywords:** Antimicrobial resistance, Colonization, Prevalence, GBS, Pregnant women

## Abstract

**Background:**

Maternal *Streptococcus agalactiae* (Group B Streptococcus, GBS) colonization rates and its antibiotic resistance patterns provide important information useful in guiding prevention strategies. There is a paucity of evidence about GBS in the Amhara National Regional State, Ethiopia.

**Objective:**

To determine colonization prevalence, associated risk factors, and antibiotics resistance including inducible clindamycin resistance patterns of GBS among Ethiopian pregnant women.

**Methods:**

A prospective cross-sectional study was conducted from 1st December 2016 to 30th November 2017 at the University of Gondar Referral hospital delivery ward. Combined recto-vaginal swabs were collected from 385 pregnant women and analyzed at the University of Gondar Bacteriology Laboratory by using LIM broth and 5% defibrinated sheep blood agar culture methods. Isolates were identified by using colony morphology, gram reaction, hemolysis, and CAMP test. Antibiotic susceptibility test was done using the disc diffusion method. Double disc diffusion method was used to identify inducible clindamycin resistance isolates. Data were analyzed by SPSS version 20 software. *p *≤ 0.05 was considered as statistically significant.

**Results:**

The overall prevalence of maternal GBS colonization was 25.5% (95% CI 21–29.5%). Experiencing meconium stained amniotic fluid (AOR = 3.018, 95% CI 1.225, 7.437), and longer duration of premature rupture of membrane (AOR = 1.897, 95% CI 1.014, 3.417) were statistically significant to maternal colonization. Furthermore, GBS resistant to 0 (8.2%), 1 (25.5%) and 3 (39.8%) or more antibiotics were identified. A D-test showed 15.2% inducible clindamycin resistant GBS. Constitutive macrolide lincosamide–streptogramin_B_, L-, and M-phenotypes were also detected.

**Conclusions:**

Maternal GBS colonization rate in this study was higher compared to the previous reports in Ethiopia. This much prevalence and antibiotics resistance results are the clue to which attention shall be given to this bacterium during management of pregnant women and the newborns.

## Introduction

*Streptococcus agalactiae* (Group B Streptococcus; GBS) is a leading cause of neonatal infections worldwide. Mother to child transmission occurs mostly during peripartum period. Evidence on maternal colonization prevalence remains sparse in the African settings [1], specifically in Ethiopia where few studies revealed maternal colonization ranges from 7.2% [2] to 20.9% [3].

A review of 390 articles from 85 countries with a total of 299,924 pregnant women showed that the adjusted estimate for maternal GBS colonization worldwide was 18% with regional variation like lower prevalence in Southern Asia (12.5% and Eastern Asia 11%) [4]. The maternal colonization prevalence among the African studies was also reported as 12% in Kenya [5] to 48.2% in South Africa [6]. It is ranged from 2.3% in India [7] to 40.8% in Italy [8]. Another review of 21 studies, including 24,093 women from 13 European countries indicated that the colonization prevalence varies from 6.5% in Turkey to 36% in Denmark [9]. One global estimate study revealed that there were 33,000 cases of invasive GBS disease in pregnant or postpartum women, and 57,000 fetal infections/stillbirths. About 3.5 million preterm births may be owing to GBS, and Africa accounted for 54% of estimated cases and 65% of all fetal/infant deaths [10]. Recto-vaginal colonization of pregnant women with GBS is a major risk factor for neonatal colonization and it has to be noted that 21–35% the GBS has recovered from the recto-vaginal samples of women of reproductive age [11–13]. Maternal intrapartum antibiotic prophylaxis (IAP) is one of the most effective means to reduce early neonatal GBS diseases [1] though it has no effect on late onset infant diseases caused by GBS. Despite this, provision of IAP is not practiced at the University of Gondar Referral hospital particularly, and in Ethiopia generally. Thus, knowing of the status of maternal colonization, mainly in low-income courtiers like Ethiopia, is useful to device the preventive strategies.

Furthermore, there is a concern about the rise of antibiotic resistances among GBS isolates so that doing antibiotic susceptibility tests of such isolates before prescription is crucial [14] to test its susceptibility pattern. Antibiotic susceptibility testing (AST) of GBS isolates in South Africa depicted 100% sensitive to penicillin, vancomycin and high level gentamicin (120 µg). However, 21.1% were resistant to erythromycin and 17.2% to clindamycin. Of these, 69% had constitutive macrolide, lincosamide–streptogramin_B_ (MLS_B_), and 17.4% had inducible MLS_B_. In addition, the M- and L-phenotypes were present in 6.8% each [15]. Of the 1160 isolates in Australia, 6.4% revealed erythromycin resistance and 4.2% to clindamycin, and 53% of the erythromycin resistant isolates showed resistance to clindamycin [16]. Certain studies in Ethiopia indicated 36.4% to 17/22 (77.3%) resistance to penicillin; 9.1% to 18.2% to clindamycin, and 4.5% to 22.6% to erythromycin [2, 17].

Maternal colonization prevalence, associated risk factors and its antimicrobial resistance patterns including inducible clindamycin resistance provide important information useful in devising prevention strategies in the area. Thus, the current study was aimed to determine maternal colonization prevalence, associated risk factors, and resistance patterns of GBS to the ten commonly prescribed antibiotics.

## Materials and methods

### Study setting

The study was conducted at the University of Gondar Referral hospital, Northwest Ethiopia. It is one of the oldest hospitals in Ethiopia located 737 km away from Addis Ababa, Capital of Ethiopia. The hospital serves more than 5 million people and it has about 450 to 600 pregnant women admission services for delivery per month with two maternities (M1 and M2). During the study period, no routine screening of pregnant women and provision of IAP has been established in the hospital.

### Study design

An institution based prospective cross-sectional study was conducted from 1st December 2016 to 30th November 2017.

### Source population and study population

All pregnant women who attended at the University of Gondar Comprehensive Specialized hospital, Northwest Ethiopia, were the source population. Pregnant women who came for delivery at the maternity ward of the hospital with gestational age of ≥ 35 weeks were the study population.

### Inclusion criteria

Pregnant women, who consented for participation in the study, did not use vaginal cream, lubricants or traditional sterilizer (vinegar) and antibiotics in the last 2 weeks prior data collection; and those who were not in emergency room, not severely ill, mentally stable and had singleton were included in the study.

### Sample size determination

The sample size was calculated by using the single population proportion estimation formula by taking 20.9% prevalence (p) of maternal colonization with GBS [3].

$$n = \frac{{z_{{\alpha /2}}^{2} \times p(1 - P)}}{{d^{2} }}$$

where; n = sample size, p = prevalence of neonatal colonization with GBS in Ethiopia (p = 50%), d = maximum allowable error (margin of error) = 0.05, Z = value of standard normal distribution (Z-statistic) at 95% confidence level (z = 1.96) and it became 254 pregnant women; however, to increase the precision/validity of the findings, the sample size was increased to 385 by taking p = 50%.

### Data collection

Socio-demographic and biological data were collected from each pregnant woman who delivered at ≥ 35 weeks of gestation at the maternity ward in the hospital until the pre-determined sample size was reached.

### Data collection tools

#### Questionnaire

A pretested questionnaire was used to collect data for assessment of socio-demography and to investigate the associated risk factors to maternal colonization with GBS. The questionnaire was prepared in English using published studies and translated into the local language (Amharic). Once data were collected, responses of each questionnaire were re-translated into English for analysis and report.

#### Biological Specimen collection and culture processing

A combined recto-vaginal swab was collected at the point of labor (before delivery) by using a sterile cotton applicator swab. Here, the lower third vagina was brushed followed by the rectal swab by the trained midwives following universal precautions [1] and was transported to the University of Gondar bacteriology laboratory within 2 to 4 h by using Amies transport medium and analyzed by following the methods described in the CDC and CLSI guidelines [1, 18]. Swabs were placed in Todd-Hewitt selective enrichment broth supplemented with colistin (10 µg/ml) and nalidixic acid (15 µg/ml) (Cart Roth GmbH + Co. KG-Schoemperlensrr. 3-5-D-76185 Karlsruhe, Germany). The inoculated selective medium was incubated at 37 °C for 24 h. Growth (turbidity) was sub-cultured onto 5% defibrinated sheep-blood agar (Oxoid, UK) and incubated for 24 h at 37 °C in 5% CO_2_ atmosphere. All suspected colonies (with narrow hemolysis) were sub-cultured on nutrient agar and subjected to gram stain and catalase test. All gram positive cocci and catalase negative isolates were tested for CAMP factor for presumptive identification.

#### CAMP (Christie–Atkins–Munch–Petersen) test

CAMP test was used to differentiate GBS (CAMP positive) from *Streptococcus pyogene* (beta-hemolytic CAMP negative) by inoculating the known *Staphylococcus aureus* onto 5% defibrinated sheep blood agar down the center of the plate with a wire loop. Group B Streptococcus (test bacterium) was then streaked in a straight line perpendicular to the *S. aureus* within 2 mm far. The plate was then incubated at 35 °C for 24 h. A positive CAMP result was indicated by an arrowhead-shaped enhanced zone of beta-hemolysis in the area between the test organism and *S. aureus* with the arrow-point towards the *S. aureus* streak. The CAMP test positive colonies were presumptively considered as GBS.

### Antimicrobial resistance testing of *Streptococcus agalactiae*

All GBS identified were tested against ten antibiotics (Oxoid, Basingstoke, UK): penicillin G (P, 10IU), ampicillin (AMP, 10 µg), clindamycin (CLY, 2 µg), erythromycin (E, 15 µg), chloramphenicol (C, 30 µg), ciprofloxacin (CIP, 5 µg), ceftriaxone (CRO, 30 µg), vancomycin (VA, 30 µg), azithromycin (AZM, 15 µg, and tetracycline (TE, 30 µg) on 5% sheep blood containing Mueller–Hinton agar according to the Kirby–Bauer method (disk diffusion) following the CLSI criteria. Bacterial inocula were prepared by suspending 4 to 5 freshly grown GBS colonies in 3–5 ml sterile physiological saline and turbidity was adjusted to a 0.5 McFarland standard [18] used as a reference to adjust the bacterial suspension for antibiotic susceptibility test. Sterile cotton swab was dipped and rotated several times, and was pressed against wall of the test tube. It was then swabbed over the entire surface of the Muller–Hinton agar containing 5% defibrinated sheep blood. Then, antibiotic impregnated paper disks were placed on the plate and incubated in 5% CO_2_ atmosphere at 37 °C for 24 h. Zone of inhibition around antibiotic disks was measured by calibrated ruler and interpreted as sensitive, intermediate or resistant by using standard chart [18].

### Detection of inducible clindamycin resistant *Streptococcus agalactiae*

Clindamycin and erythromycin susceptibility and determination of different phenotypes of macrolide–lincosamide–streptogramin_B_ (MLS_B_) resistance were performed by double-disk diffusion method on Mueller–Hinton agar (Biokar, France) containing 5% sheep blood [18, 19]. Erythromycin (15 µg) and clindamycin (2 µg) disks (Oxoid) were placed 12 mm apart edge to edge [19]. After 24 h of incubation at 37 °C, blunting of clindamycin inhibition zone proximal to erythromycin disk was taken as inducible MLS_B_ resistance. Resistance to both clindamycin and erythromycin with no blunting of clindamycin inhibition zone indicated constitutive resistance. M-phenotype (efflux mechanism) was characterized by resistance to erythromycin but susceptibility to clindamycin with no blunting of inhibition zone around clindamycin disk. Eventually, susceptible to erythromycin but resistance to clindamycin was referred to as L-phenotype [19].

### Quality control

Pre-test was done to check the questionnaire and the protocol for GBS identification. Data cleaning was done every day. *Streptococcus agalactiae* (ATCC 12386), *Enterococcus faecalis* (ATCC 29212); *Streptococcus pyogenes* (ATCC 19615), *Staphylococcus aureus* (ATCC 29213) and *Escherichia coli* (ATCC 25922) were used as quality control in this study.

### Definitions of terms

Early onset disease: Diseases which appear from birth to 6th completed days.

Late onset disease: Diseases which appear in infants between the 1st week and 89 days of age.

Premature rupture of membrane (PROM), or pre-labor rupture of membrane: a rupture of membrane (breakage of the amniotic sac), commonly called breaking of the mother’s water(s), more than 1 h before the onset of labor.

Preterm delivery: delivery before 37 completed weeks of pregnancy.

### Data analysis and interpretation

Data were entered into excel spread sheet and exported to SPSS 20 (Chicago, IL, USA) and analyzed. Descriptive statistics aimed to summarize the study participants’ characteristics across the outcome variable was used. Association between the outcome variable (colonization of pregnant women with GBS) and each independent variable (demography and clinical factors) was analyzed using bi-variable and multi-variable logistic regression model. All the variables were entered into the multivariable logistic regression using backward LR method to control the confounding effect. Explanatory variables which had significant association with the materna GBS colonization at a *p* value ≤ 0.2 in the bi-variable logistic regression model were entered to the multivariable logistic regression model to identify the risk factors associated to the colonization of pregnant women with GBS. Assumption of goodness of the model was checked by Hosmer–lemeshow test (p = 0.828). Association between the outcome and the independent variables was calculated by using the adjusted odds ratio at a p-value ≤ 0.05 and 95% confidence interval.

## Results

### Demography, obstetric characteristics and maternal colonization

Among the total of 385 pregnant women, with ≥ 35 gestational week of pregnancy, participated in our study, 77.1% were below the age of 25 years old, 82.9% were urban dwellers, and 74.3% were house wives. Almost half (49.1%) of the participants were nullipara, 67.8% had four or more times of ANC follow up, 2.3%, 4.8% and 8.6% had history of neonatal death, still birth and abortion respectively (Table 1).

**Table 1:** Maternal GBS colonization by demography and obstetric characteristics including multivariable analysis, Northwest Ethiopia (n = 385)

Socio-demography	Classification	GBS+, n′ = 98, n (%)	GBS−, n = 287, n (%)	COR^a^; 95% CI	AOR^b^, 95% CI^c^	p-value
Age (years) median = 25 years	< 25	74 (24.9)	223 (75.1)	1	–	–
	≥ 25	24 (27.3)	64 (72.7)	1.130 (0.660, 1.935)	–	–
Residence	Urban	77 (24.1)	242 (75.9)	1	–	–
	Rural	21 (31.8)	45 (68.2)	0.626 (0.353, 1.109)	–	–
Education	Illiterate	30 (34.5)	57 (65.5)	0.550 (0.212, 1.426)	–	–
	Primary	21 (16.0)	110 (84.0)	1.689 (0.640, 4.459)	–	–
	Secondary	40 (29.2)	97 (70.8)	0.738 (0.293, 1.857)	–	–
	Tertiary	7 (23.3)	23 (76.7)	1	–	–
Occupation	House wife	72 (25.2)	214 (74.8)	1	–	–
	Employed	21 (28.4)	53 (71.6)	0.849 (0.479, 1.504)	–	–
	Others^d^	5 (20.0)	20 (80.0)	1.346 (0.487, 3.716)	–	–
Gestational age (week)	< 37	1 (33.3)	2 (66.7)	0.681 (0.061, 7.590)	–	–
	≥ 37	97 (25.4)	285 (74.6)	1	–	–
Parity	Multipara	54 (27.6)	142 (72.4)	0.798 (0.504, 1.265)	–	–
	Nullipara	44 (23.3)	145 (76.7)	1	–	–
History of still birth	No	94 (25.6)	273 (74.4)	1	–	–
	Yes	4 (22.2)	14 (77.8)	1.205 (0.387, 3.752)	–	–
History of abortion	No	92 (26.1)	260 (73.9)	1	–	–
	Yes	6 (18.2)	27 (81.8)	1.592 (0.637, 3.980)	–	–
History of neonatal death	No	93 (24.7)	283 (75.3)	1	–	–
	Yes	5 (55.6)	4 (44.4)	0.263 (0.063, 1.000)	–	–
Gravidity	Primigravida	42 (22.8)	142 (77.2)	1	–	–
	Multigravida	56 (27.9)	145 (72.1)	0.766 (0.482, 1.216)	–	–
Antenatal care (ANC) visit	0–3	31 (25.0)	93 (75.0)	0.973 (0.596, 1.588)	–	–
	4–5	67 (25.7)	194 (74.3)	1	–	–
Contraceptive use	No	12 (17.6)	56 (82.4)	1	–	–
	Yes	86 (27.1)	231 (72.9)	0.576 (0.294, 1.126)	–	–
Meconium stained amniotic fluid	No	92 (27.4)	244 (72.6)	1	1	
	Yes	6 (12.2)	43 (87.8)	2.229 (0.966, 5.139)	3.018 (1.225, 7.437)	*0.016*
History of preterm delivery	No	95 (25.1)	284 (74.9)	1	–	–
	Yes	3 (50.0)	3 (50.0)	0.337 (0.067, 1.697)	–	–
Chronic illness at current pregnancy	No	90 (24.8)	273 (75.2)	1	–	–
	Yes	8 (36.4)	14 (63.6)	0.905 (0.344, 2.382)	–	–
ROM^e^	≤ 1 h	74 (27.6)	194 (72.4)	1	1	
	> 1 h	24 (21.1)	90 (78.9)	1.313 (0.781, 2.378)	1.897 (1.014, 3.417)	*0.033*
Duration of labor (hour)	4–12	81 (25.6)	235 (74.4)	1	–	–
	13–24	17 (24.6)	52 (75.4)	1.054 (0.577, 1.927)	–	–
HIV status	Negative	94 (25.3)	278 (74.7)	1	–	–
	Positive	4 (30.8)	9 (69.2)	0.761 (0.229, 2.528)	–	–

Italic values indicate significance of p-value (p < 0.05)

^a^Crude odds ratio

^b^Adjusted odds ratio

^c^Confidence interval

^d^Student, daily laborer

^e^Rupture of membrane

The overall prevalence of colonization of pregnant women with GBS at the time of labour was 98/385 [25.5% (95% CI 21–29.5)]. Those pregnant women with < 25 years old (19.22%), urban dwellers (20.0%), house wife (18.7%), multiparity (15.21%), and gestational age of pregnancy ≥ 37 weeks (25.19%) had higher prevalence of maternal colonization than their counterparts. A multi-variable logistic regression analysis showed that those pregnant women who experienced meconium stained amniotic fluid were 3 times (AOR = 3.018, 95% CI 1.225, 7.437) more likely to have increased risk of colonization than their counterparts. In addition, the pregnant women who had longer duration of premature rupture of membrane/ROM were 2 times (AOR = 1.897, 95% CI 1.014, 3.417) more likely to have an increased risk of being colonized than those who had shorter duration of premature ROM (Table 1).

### Antimicrobial resistance profiles of *Streptococcus agalactiae*

Among the 98 GBS isolates identified in our study, 10 (10.2%) and 9 (9.2%) of the isolates showed resistance to penicillin and ampicillin. The highest resistance was observed to tetracycline 72 (73.4%) followed by 31 (31.6%) to ceftriaxone, 26 (26.5%) to erythromycin, and 21 (21.4%) to clindamycin excluding inducible resistance (Tables 2, 3). *Streptococcus agalactiae* resistance to 0 (8.2%), 1 (25.5%) and 3 (39.8%) or more antibiotics were also identified.

**Table 2:** Antibiotic resistance profiles for Recto-vaginal colonizing group B streptococcal isolates, Northwest Ethiopia

Antibiotics	GBS isolates (n = 98)		
	Susceptible, n (%)	Intermediate, n (%)	Resistant, n (%)
Penicillin	88 (89.8)	0 (0.0)	10 (10.2)
Ampicillin	89 (90.8)	0 (0.0)	9 (9.2)
Erythromycin	66 (67.3)	6 (6.1)	26 (26.5)
Clindamycin	72 (73.5)	5 (5.1)	21 (21.4)^a^
Azithromycin	76 (77.6)	3 (3.1)	19 (19.4)
Vancomycin	82 (83.7)	0 (0.0)	16 (16.3)
Ceftriaxone	67 (68.4)	0 (0.0)	31 (31.6)
Ciprofloxacin	84 (85.7)	0 (0.0)	14 (14.3)
Chloramphenicol	83 (84.8)	4 (4.1)	11 (11.2)
Tetracycline	19 (19.4)	7 (7.1)	72 (73.4)

^a^Excluding the inducible clindamycin resistant isolates (iMLS_B_)

**Table 3:** Macrolide, lincosamide–streptogramin B (MLSB) and inducible clindamycine resistance GBS isolates, Northwest Ethiopia (n = 46)

Double disc diffusion								
GBS phenotypes^a^	Erythromycin (n)			Clindamycin (n)			Total (n = 46)	Percent (%)
	R	I	S	R	I	S		
Constitutive macrolide, lincosamide–streptogramin B (cMLS_B_)	10	2	–	9	3	–	12	26.1
inducible macrolide, lincosamide–streptogramin B (iMLS_B_)	6	1	–	–	–	7	7	15.2
M-phenotype	10	3	–	–	–	13	13	28.3
L-phenotype	–	–	14	12	2	–	14	30.4
D-shape positive	6	1	0	–	–	7	7	15.2
D-shape negative	–	–	–	–	–	–	39	84.8

^a^CLSI disk diffusion breakpoints [18]. Erythromycin: ≥ 21 mm, susceptible (S); 16 to 20 mm, intermediate (I); ≤ 15 mm, resistant (R). Clindamycin: ≥ 19 mm, susceptible (S); 16 to 18 mm, intermediate (I); ≤ 15 mm, resistant (R)

### Inducible clindamycin resistance (D-shape) of *Streptococcus agalactiae*

The phenotypic analyses of 46 GBS results detected by double disc diffusion method are summarized in Table 3. Among the resistant and/or intermediate resistant isolates to erythromycin and clindamycin, 30.4% harboured L-phenotypes (lincosamide resistance); 28.3% M-phenotypes (efflux mechanism); 26.1% constitutive MLS_B_, and 15.2% inducible MLS_B_.

## Discussion

The prevalence of maternal colonization with GBS observed in our study was 25.5% (95% CI 21–29.5%). This much prevalence of maternal colonization with GBS may cause the presence of transmission of GBS from colonized pregnant women to neonates delivered from the GBS positive mothers in the study area and is useful to devise preventive strategies like IAP which is not yet practiced at the University of Gondar Referral hospital as well as in Ethiopia. Such a result is consistent with an overall recent estimate of maternal colonization reported in Africa (21.3%), in sub-regions of Africa such as North Africa (22.9%), Middle Africa (23.9%, and Southern Africa (28.9%) [4]. Our result is also similar with various studies conducted in different parts of the world that ranged from 20.9% in Ethiopia, Zimbabwe and Palestine [3, 20, 21] to 28.7% in Poland [22]. However, it is lower than a report South Africa (48.2%) [6]. Our study showed higher prevalence of maternal colonization with GBS than the adjusted worldwide estimates of colonization (18%) with lower prevalence in sub-regions like Southern Asia (12.5% and Eastern Asia (11%) [4]; in India, 2.3% [7] and 20% in Democratic Republic of the Congo [23]. These discrepancies might be owing to the clinical characteristics, body site sampled, and time of screening for GBS. Accessibility of pregnant women screening for GBS, IAP provision, and detection techniques employed for GBS may also attribute for this variations. The latter could be justified by the fact that some studies used 5% sheep blood agar (BAP); others used combination of enrichment selective culture media (ECM) and BAP, or ECM/PCR (polymerase chain reaction) combination, serology or CAMP test [24]. Specimen storage conditions, duration of sample transportation, use of antibiotics and antiseptic products may also cause for disparities of GBS detection among studies. Swabbing cotton tips used, epidemiological characteristics, and different study designs might be the additional contributing reason for this variability across the studies.

Knowledge about risk factors associated to maternal colonization is useful to reduce morbidity and mortality related to GBS diseases. This study, found that socio-demography (age; occupation; educational status); obstetrics (antenatal visit; gravidity; gestational age; parity; history of still birth, preterm delivery, abortion, neonatal death, contraceptive use; chronic illness at current pregnancy including HIV; and duration of labor) did not associate to the maternal colonization like other various studies reported [3, 25–28]. Studies in Greece and Gambia found that increasing number of antenatal visits [25, 29], and midwife delivery were associated with maternal colonization [29]. Similarly, a study from Thailand revealed that older maternal age and lower gestational age were found to be the risk factors for maternal colonization [30]. Experiencing meconium stained amniotic fluid and length of premature ROM were significantly associated factors to maternal colonization in our study. The latter finding is supported by studies done in Nigeria and India [28, 31].

*Streptococcus agalactiae* resistance to penicillin, ampiciline, vancomycin, clindamycin and other tested antimicrobials is observed in the current study. This observation may help to alert the concerned bodies to minimize empirical therapy and to establish the antimicrobial stewardship in the study area. Similarly, reported resistance patterns to penicillin are different in different studies. Certain studies done in Ethiopia revealed that resistance to penicillin is ranged from 36.4 to 77.3% [2, 17] while others in same country showed no resistance to penicillin, ampicillin and/or vancomycin [3, 32, 33]. Worldwide studies also revealed nearly absence of pencillin, ampicillin and/or vancomycin resistant isolates; for example Tanzania [34]; Ghana [35]; South Africa [15]; China [36]; Brazil [37] and United Kingdom [38]. However, a recent data in Palestine showed 38.0% penicillin and 21.0% vancomycin resistance [21]. This showed that the magnitudes of resistance seen in the literatures are inconsistent with the observations of the current study. This variation might be due to the inadequacy of laboratory facilities and laboratory difficulties to do antibiotic resistance procedures for GBS in developing countries lead the physicians to treat patients empirically.

Significant number of GBS isolates showed erythromycin and clindamycin resistance in the current study. This may decrease the options for prophylaxis in pregnant women who are allergic to penicillin. A few studies conducted in Ethiopia demonstrated that 22.7% GBS were resistant to erythromycin and 17.6% to 18.2% to clindamycin [17]. A South African study revealed that 21.1% of the isolates were resistant to erythromycin and 17.2% to clindamycin [15]. Another reports from Tanzania also indicated that 17.6% of GBS were resistance to clindamycin [34]; and 21.0% to clindamycin in USA [39]. These reports are in agreement with results of our study. However, we found higher resistance rate to erythromycin than the other studies conducted in same country (6.9% to 11.8%) [3, 33]; 13.0% in Tanzania [34]; 8.62% and 8.1% in Brazil [37]. Different studies reported higher resistance rates that 45.2% to 50.7% resistance to erythromycin, and 36.7% (including D-test positives) to 38.4% to clindamycin in USA [40, 41]; 25.6% to erythromycin and 54.0% to clindamycin in Korea [42]; 43.0% to erythromycin and 69.0% to clindamycin in Palestine [21]; and 66.2% to erythromycin and 54.0% to clindamycin in China [36].

The highest antibiotic resistance rate was observed to tetracycline in our study (73.4%). Similar resistance rates were reported in Tunisia (97.3%) [43]; Iran (96%) [44] and Palestine (45.0%) [21]. Resistance rates to tetracycline, ceftriaxone, erythromycin and clindamycin observed in our study corroborates the resistance data to these antibiotics reported by Lambiase et al. and Emaneini et al. [8, 44]. It might be caused by the widespread use of these antibiotics for different clinical cases that possibly leads to the emergence of antibiotic-resistance GBS.

A phenotypic analysis of the 46 erythromycin and/or clindamycin resistant/intermediate GBS in our study revealed that 30.6% harboured L-phenotypes; 28.3% M-phenotypes; 26.1% cMLS_B_, and 15.2% iMLS_B_ phenotypes. Among the erythromycin and/or clindamycin resistance isolates analyzed in South Africa, 69% had cMLS_B_ and 17.4% had iMLS_B_. The M- and L-phenotypes were present in 6.8% each [15]. A Tunisian report also showed that among erythromycin resistant isolates, 78.7%, 10% and 2.2% had cMLS_B_, iMLS_B_, and M-phenotypes respectively [43]. Studies in Iran revealed that all the erythromycin resistant GBS had cMLS_B_ [44] and 17.4% had iMLS_B_ [45]. A study in USA reported that 8% had a positive D-test, indicating inducible clindamycin resistance [40]. Another study in USA also displayed 44% cMLS_B_, 21% iMLS_B_, 28% M-, and 6% L-phenotype harbored isolates [39]. It is believed that the differences in antibiotics use, prophylaxis practice, widespread and indiscriminate use of these antibiotics in various clinical cases, variation in susceptibility test methods and/or disparities in serotypes distribution may result to regional differences in resistance rates of GBS to different antibiotics.

## Conclusion

The present study showed higher prevalence of maternal GBS colonization compared to previous Ethiopian studies. Meconium stained amniotic fluid and lengthy premature rupture of membrane were found to be the risk factors for maternal colonization. It also identified the presence of antimicrobial resistant GBS to penicillin, ampicillin, vancomycin, ceftriaxone and other tested antimicrobials. GBS with inducible clindamycin resistant were identified. Furthermore, L- M- and cMLS_B-_phenotypes harboring GBS were detected. On the bases of the findings, pregnant women screening for GBS at their late third trimester, antibiotic susceptibility test before prescription, provision of intrapartum antibiotic prophylaxis, and large scale epidemiological studies have to be put into practice in the study area.
